# Association Between Early Postpartum Maternal Fatigue and Infant Emotional Development: Protocol for a Prospective Cohort Study

**DOI:** 10.2196/97348

**Published:** 2026-11-05

**Authors:** Zimeng Xu, Jiahui Ni, Ruotong Wei, Feng Zhang

**Affiliations:** 1 School of Nursing and Rehabilitation, Nantong University, No. 18, Nursing Home Road, Chongchuan District, Nantong, Jiangsu, 226001, China, 86 13814715979

**Keywords:** postpartum maternal fatigue, infant, emotion, mother-infant attachment, oxytocin

## Abstract

**Background:**

Postpartum fatigue (PPF) is clinically defined as persistent physical and mental exhaustion emerging after childbirth that cannot be fully relieved by rest. It is distinct from postpartum depression and has a prevalence of 76.13% among Chinese women within the first postpartum week. Existing evidence suggests that maternal PPF may alter salivary oxytocin concentrations, disturb maternal caregiving behaviors, and subsequently interfere with multiple dimensions of infant emotional development, including emotional expression, emotional regulation, and emotional recognition. Objective psychophysiological measurements, such as eye-tracking and galvanic skin response, have been validated for assessing infant emotional responses. Nevertheless, most existing studies focus merely on maternal mental well-being. Local longitudinal evidence clarifying this sequential mechanistic chain remains limited, and targeted early screening and intervention strategies for PPF-affected mother-infant dyads are insufficient.

**Objective:**

This prospective cohort protocol aims to examine the independent association between maternal PPF and the emotional development of 3-month-old infants and to explore the sequential mediating roles of salivary oxytocin and mother-infant caregiving quality. The primary hypothesis is that higher PPF severity will correlate with poorer infant emotional development outcomes. The secondary hypothesis is that oxytocin levels and maternal caregiving sequentially mediate this association.

**Methods:**

We will recruit 412 mother-infant dyads with maternal PPF within 1 week postpartum. PPF severity will be measured by the Postpartum Fatigue Scale. Infant emotional development, covering expression, regulation, and recognition, will be evaluated via questionnaires and physiological assessments, including eye-tracking and galvanic skin response, at an infant age of 3 months. General analytical frameworks will be applied for hypothesis testing.

**Results:**

This study was funded and initiated in September 2025. Participant recruitment commenced in October 2025. A total of 180 mothers-infants dyads underwent eligibility screening; 146 of these dyads completed enrollment, yielding a participation rate of 81.1%. Twelve dyads withdrew before the 3-month follow-up window, and 4 dyads were lost to follow-up at the 3-month assessment. To date, 112 dyads have completed the full 3-month postpartum assessment, and the remaining participants are still under follow-up. No major protocol deviations have occurred. Data analysis of samples from participants who have completed the 3-month assessments is ongoing. According to the prespecified timeline, full sample collection is projected to be completed by December 2027, and formal data analysis will begin shortly thereafter. The study is progressing steadily in accordance with preset schedules. Full dataset analysis and submission of the final manuscript are anticipated in early 2028.

**Conclusions:**

This cohort will generate longitudinal evidence describing links between maternal PPF and early infant emotional development in Chinese postpartum populations. The findings may help identify high-risk mother-infant subgroups and provide real-world evidence to develop targeted early screening and dyadic support strategies for postpartum mothers and their infants.

## Introduction

Postpartum fatigue (PPF) is one of the 5 most common symptoms occurring in the perinatal period that has received considerable attention from researchers across the globe due to its common occurrence and potential impact [1]. This tiredness often starts immediately after childbirth and tends to peak in the first 36 hours [2]. After that, it may persist for several weeks or months after delivery as an overwhelming fatigue [3]. Fatigue is both a subjective and objective feeling of tiredness or loss of energy experienced by an individual [4].

Epidemiological studies have shown a high prevalence of PPF. In a cohort of first-time mothers from China, approximately 76.13% experience some level of fatigue during the first week after childbirth [5]. Among these, 38.81% report moderate fatigue, while 7.46% experienced severe fatigue [6]. The research further highlights the extremely common occurrence of PPF during the initial days postdelivery [7]. It shows that 11.4% of mothers reported experiencing severe fatigue after 3 months postdelivery [8]. Interestingly, 66% of women still feel exhausted 18 months after giving birth [2]. Rates of PPF noted in the United Kingdom are 38.8% at 10 days, 27.1% at 1 month, and 11.4% at 3 months postpartum. Furthermore, among Turkish women, 77.5% may suffer within the first 6 weeks, while 33.9% continue to feel fatigue 1 year after birth [9].

In the present study, PPF is measured using the Postpartum Fatigue Scale (PFS) [5]. This instrument evaluates multidimensional fatigue manifestations across physical, mental-cognitive, and affective dimensions. A total PFS score greater than 10 is adopted as the operational cutoff for identifying PPF. Fatigue severity is further stratified according to scale scoring criteria: scores of 11 to 14 indicate mild fatigue, 15 to 20 indicate moderate fatigue, and 21 to 40 indicate severe fatigue. This operational definition distinguishes PPF from general everyday tiredness and chronic psychological stress.

Notably, this condition is not merely fatigue. It represents a multifaceted symptom that develops following the injury [10]. Indeed, several factors contribute, such as the body’s need for recovery, the psychological adjustments required, and the difficulties in looking after a newborn [11].

The emotional development of infants and toddlers is a process that changes from simple reflexes to complex feelings over time [12]. This development refers to feelings and how they are expressed, managed, and recognized [13]. Newborns may be able to display emotions a day or 2 after birth [14]. At around 7 to 8 weeks, infants can differentiate social emotions [15]. When infants reach 3 months of age, they have developed to the point where they have made substantial advances in their ability to convey emotion [16]. During this time, positive interactions with caregivers are important for providing emotional security to the children, which will help the child regulate emotion and develop social skills later in life [17].

Persistent physiological strain caused by PPF may induce neuroendocrine responses similar to chronic stress. These responses disrupt the hypothalamic-pituitary neuroendocrine pathway and inhibit the synthesis and release of oxytocin from oxytocinergic neurons within the hypothalamic supraoptic and paraventricular nuclei, resulting in reduced salivary oxytocin concentrations in mothers [18].

Oxytocin positively facilitates maternal caregiving behaviors and mother-infant attachment by modulating neural activity in emotion-regulating brain regions, including the nucleus accumbens, anterior cingulate cortex, amygdala, and ventral hippocampus [19]. Insufficient maternal oxytocin secretion reduces maternal sensitivity to infant cues, decreases synchronous dyadic interactions, and impairs the quality of mother-infant attachment [20]. Low-quality mother-infant interactions disrupt biobehavioral synchrony within dyads. Such interaction impairment hinders the normative maturation of infant brain regions responsible for emotional processing, including the amygdala and hippocampus, and increases baseline sympathetic arousal. Meanwhile, it deprives infants of essential interactive feedback that supports the development of emotional expression, emotional regulation, and emotional recognition abilities [21]. Additionally, inadequate maternal oxytocin release during mother-infant contact simultaneously reduces peripheral salivary oxytocin levels in infants and further exacerbates developmental deficits in infant emotion, ultimately leading to 3 adverse developmental manifestations, including insufficient positive emotional expression, impaired capacity to modulate negative emotions, and a blunted ability to recognize facial emotional cues. This establishes a sequential transmission pathway in which maternal PPF sequentially shapes infants’ 3D emotional development via oxytocin levels and maternal caregiving behaviors [22].

Despite established links between maternal mental health and child development, critical knowledge gaps remain. First, existing research on PPF has predominantly focused on maternal physical and mental health outcomes, with limited longitudinal evidence examining its causal impact on infant emotional development, particularly in the early postpartum period. Second, the underlying mechanisms linking PPF to infant emotional development remain poorly characterized; few studies have systematically explored the chain-mediating roles of oxytocin and mother-infant attachment, thereby limiting understanding of the biological and behavioral pathways. To address these gaps, this prospective cohort study aims to examine the independent association between maternal PPF and the emotional development of 3-month-old infants and to investigate the mediating roles of salivary oxytocin and mother-infant attachment in this association.

## Methods

### Design

This is an observational study. The study was designed to test 2 prespecified hypotheses: (1) higher severity of maternal PPF is independently associated with poorer emotional developmental outcomes in 3-month-old infants; and (2) salivary oxytocin levels and mother-infant attachment quality sequentially mediate the association between maternal PPF and infant emotional development.

This protocol is reported in accordance with the STROBE (Strengthening the Reporting of Observational Studies in Epidemiology) guidelines for cohort studies. The completed STROBE checklist is provided with this submission (Checklist 1).

### Technical Route

Technical route for the effect of maternal PPF on offspring emotional development (Figure 1).

**Figure 1.**
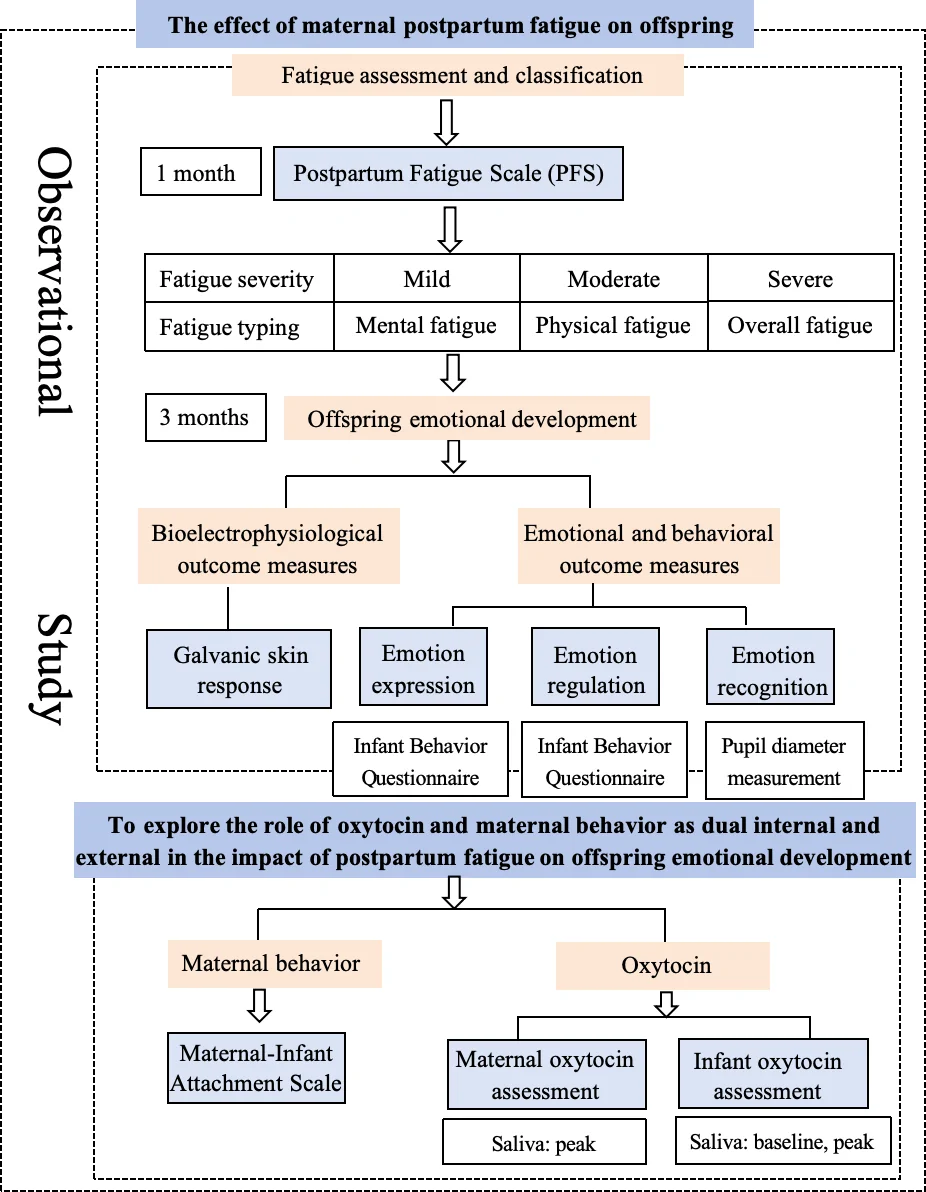
Study flow chart and technical route for the effect of maternal postpartum fatigue on offspring emotional development.

### Setting

From October 2025 to June 2026, we will recruit women who recently gave birth, specifically those 1 week postpartum, from the Nantong Maternity and Child Health Hospital in China. Each month, approximately 300 new mothers attend routine health checks at the outpatient center, providing a sufficient number of candidates meeting the study’s eligibility criteria.

### Target Population

Mothers eligible for the program must be at least 18 years of age and must have given birth at full term (gestational age of 37 wk or more). Participants of the study must have voluntarily agreed to participate within 1 week of delivery and have signed the consent form. Infants must not have any congenital conditions, neurodevelopmental conditions, or major physical illnesses. In order to assist with questionnaires and follow-up assessments, mothers should possess basic communication skills.

Exclusion criteria are as follows:

- Long-term, regular mother-infant separation (≥8 h per d), as reduced daily mother-infant interaction would attenuate the hypothesized causal pathway between maternal fatigue and infant development and introduce substantial misclassification of exposure effects [23].
- Individuals with clinically diagnosed severe mental disorders before pregnancy or during the prenatal period, including schizophrenia, bipolar affective disorder, psychotic depression, and organic mental disorders. Preexisting severe psychiatric conditions may interfere with the evaluation of fatigue that emerges after childbirth and obscure its unique predictive effect on infant emotional development [24].
- Severe maternal chronic diseases (diabetes, hypertension, thyroid dysfunction, and genetic metabolic diseases), as these conditions directly alter maternal inflammatory and neuroendocrine status, including oxytocin regulation, and are independently associated with both fatigue severity and infant developmental outcomes [25].
- Infants with congenital malformations, birth asphyxia, or severe neonatal diseases, as such neonatal pathological conditions are strong independent predictors of early emotional developmental delay and would distort the observed association [26].
- Maternal use of medications affecting oxytocin levels (eg, oxytocin receptor antagonists) during pregnancy or postpartum, as these agents directly interfere with the key mediating variable in the proposed mechanistic pathway [27].
- New-onset severe postpartum complications (eg, massive postpartum hemorrhage or severe infection) occurring during follow-up, as these acute events drastically alter the maternal fatigue trajectory and caregiving capacity and represent extreme outliers unrepresentative of typical PPF [28].

### Sample Size

#### Sample Size Estimation Based on Correlation Analysis

Previously conducted studies indicated that the PPF occurrence among Chinese women was 76.13%. The sample size was calculated using the formula [(Z_α/2_ + Z_β_)/Z_r_]² + 3, where Z_r_=0.5 × ln[(1+r)/(1–r)]. The important parameters are α=.05, β=.1, Z_α/2_=1.96, and *r*=0.2. These parameters represent the significance level, type  II error rate, the critical value, and correlation coefficient, respectively. The expected correlation coefficient *r*=0.2 was derived from published prospective cohort studies with reported correlation coefficients ranging from 0.18 to 0.26. An estimated minimum sample size of 259 cases resulted from this. With a 20% anticipated loss to follow-up, the final minimum sample size is 324 mother-infant dyads [29].

#### Sample Size Requirement for Multiple Linear Regression

For multivariate linear regression, criteria proposed by Green [30] were applied to ensure adequate statistical power for both overall model testing and evaluation of individual covariate effects. When 30 confounding covariates are included in the regression model (m=30), the recommended minimum sample size ranges from 134 to 290 valid participants, according to these criteria. The higher threshold of 290 was adopted to guarantee sufficient power for the overall regression model. The widely accepted empirical standard of 10 participants per covariate was only regarded as a secondary reference because it may yield biased statistical power estimates under moderate effect sizes [30].

#### Sample Size Requirement for Structural Equation Modeling and Bootstrap Mediation Analysis

Structural equation modeling (SEM) with bias-corrected bootstrap mediation will be applied to test sequential mediating pathways. Our considerations regarding sample size for mediation analysis draw on the literature survey reported by Fritz and MacKinnon [31]. According to their survey of existing mediation studies using SEM, the median sample size was 239.5 (IQR 152–413). Considering potential measurement error within observational cohort data and the large set of 30 confounding covariates adjusted in our model, we adopted a conservative empirical minimum of 200 dyads of mothers and infants to ensure stable model convergence and reliable bootstrap mediation inference [31].

After comparing the results from the 3 calculation approaches above, the largest sample size (324, adjusted for 20% attrition) was selected as our baseline requirement. To further accommodate subgroup-stratified analyses by fatigue severity and dimensions, we finally set the target enrollment number to 412 mother-infant dyads to ensure adequate statistical power for restricted cubic spline models, mediation analysis, and SEM.

### Procedures

Data were collected at 2 time points: baseline (within 7 d postpartum) and 3-month follow-up (corrected age).

#### First Assessment (Within 1 Wk Postpartum)

After eligible participants are identified in the maternity ward and postnatal clinic, individuals who meet the inclusion criteria and provide informed consent will be included in the study. Standardized questionnaires and medical record reviews will be used to collect information on demographics, obstetric history, and postpartum maternal fatigue. Maternal PPF will be measured using the PFS. This scale stratifies maternal fatigue into mild, moderate, and severe grades and covers 2 dimensions, including mental fatigue and physical fatigue.

#### Second Assessment (3 Mo Postpartum)

Mother-infant dyads completed the follow-up assessment. The evaluation includes maternal indicators, infant indicators, and saliva sample collection, detailed below. First, infant emotional behavior across 3 dimensions (emotional expression, regulation, and recognition) was assessed by combining physiological indicators, including skin conductance response and pupil diameter variation, with the Infant Behavior Questionnaire-Revised Very Short Form (IBQ-R VSF). Second, maternal caregiving behaviors will be evaluated using the Chinese Maternal Postnatal Attachment Scale. Salivary oxytocin concentrations of mothers and infants will be measured to explore the mechanistic roles of maternal caregiving behaviors and the oxytocin system in the association between maternal PPF and infant emotional development.

Once the data collection is complete, the information will be organized, classified, and entered into the database. Appropriate statistical methods will be employed to examine the associations between postnatal fatigue and infant emotional growth, as well as the mediating effects of oxytocin and maternal behaviors.

Table 1 summarizes all assessment measures, their timing, and their corresponding analytical purposes to explicitly link data collection to the statistical analysis plan.

**Table 1.** Overview of study assessments and data collection schedule.

Time point, data source, and variables	Assessment tool	Data collection	Role of variable
Baseline (postpartum week 1)			
Mothers			
Primary exposure: postpartum fatigue	Chinese Postpartum Fatigue Scale (PFS) [5]	Self-report	Exposure
Covariates: sociodemographics and obstetric factors	Structured questionnaire	Interviewer-administered	Confounder adjustment
Covariates: maternal depressive symptoms	Edinburgh Postnatal Depression Scale (EPDS) [32]	Self-report	Confounder adjustment
Covariates: maternal anxiety symptoms	Generalized Anxiety Disorder-7 (GAD-7)	Self-report	Confounder adjustment
Covariates: postpartum sleep quality	Pittsburgh Sleep Quality Index (PSQI)	Self-report	Confounder adjustment
Covariates: family support	Perceived Social Support Scale (PSSS, family-support subscale)	Self-report	Confounder adjustment
Covariates: parenting stress	Chinese short-form Parenting Stress Index (PSI-SF)	Self-report	Confounder adjustment
Covariates: family rearing environment	0‐1-Year-Old Child Family Rearing Environment Scale [33]	Caregiver report	Confounder adjustment
Fathers			
Covariates: paternal sociodemographic characteristics	Structured questionnaire	Interviewer-administered	Confounder adjustment
Covariates: paternal mental health (anxiety symptoms)	Generalized Anxiety Disorder-7 (GAD-7)	Self-report	Confounder adjustment
Infant			
Covariates: infant sleep status	Brief Infant Sleep Questionnaire (BISQ) [34]	Caregiver report	Confounder adjustment
Covariates: infant temperament	Early Infancy Temperament Questionnaire (EITQ)	Caregiver report	Confounder adjustment
3-month follow-up			
Infants			
Primary outcome: infant emotional development	IBQ-R^a^ Very Short Form [35] (emotional expression, regulation)	Caregiver report	Outcomes
Primary outcome: infant emotional recognition	Tobii X30-120 eye tracker (pupil diameter) [36]	Physiological	Outcomes
Primary outcome: infant emotional arousal	Shimmer3 GSR^b^ device [37]	Physiological	Outcomes
Mothers			
Behavioral mediator: mother-infant attachment	Chinese Maternal Postnatal Attachment Scale (CMPAS) [38]	Self-report	Mediator
Mothers/infants			
Biological mediator: maternal/infant oxytocin [39]	Saliva sample+ELISA^c^	Biomarker	Mediator

a IBQ-R: Infant Behavior Questionnaire-Revised.

b GSR: galvanic skin response.

c ELISA: enzyme-linked immunosorbent assay.

### Exposure

Maternal PPF was assessed using the Chinese version of the PFS, translated and validated by Qian et al [40,41]. This 10-item self-report scale comprises 2 dimensions: physical fatigue (6 items) and mental fatigue (4 items). Items are rated on a 4-point Likert scale, with total scores ranging from 10 to 40; higher scores indicate more severe fatigue. For operational classification, severity is stratified as no fatigue (10 points), mild (11‐14 points), moderate (15‐20 points), and severe (21‐40 points). The Chinese version has excellent internal consistency (Cronbach α=0.818) and is the most widely used tool for PPF assessment in Chinese populations.

### Outcome

#### Overview

The primary outcome is infant emotional developmental level at 3 months of corrected age, assessed across 4 core domains: emotional expression, emotional regulation, emotional recognition, and emotional electrophysiological response. This outcome was selected to directly test our core hypothesis that higher maternal PPF is associated with poorer infant emotional development. Salivary oxytocin concentration and mother-infant attachment quality are designated as secondary mediating outcomes. They were chosen based on current neuroendocrine and developmental evidence, given their sequential intermediate roles in the hypothesized causal pathway linking PPF to infant emotional outcomes.

#### Emotional Expression

The IBQ-R VSF was initially developed by Rothbart in 1981 and underwent multiple subsequent revisions [42]. The very short form, containing 37 items distributed across 3 subscales (Negative Emotionality, Positive Affectivity, and Regulatory Capacity), was developed by Putnam research team [43]. Translated into numerous languages, this instrument has been widely used to assess infant temperament worldwide. In the present study, 25 items from the Negative Emotionality and Positive Affectivity subscales are used to evaluate infants’ emotional behaviors and physiological stress responses. All items are rated on a 7-point scale with an additional “not applicable” option and are completed by infant caregivers. The Chinese version exhibits good reliability and validity, with Cronbach α exceeding 0.70 for most subscales [1].

#### Emotional Regulation

Infant emotional regulation is evaluated using the 12-item regulatory capacity subscale from the IBQ-R VSF [44]. The scale demonstrates satisfactory reliability and validity, with Cronbach α>0.70 for most subscales. Each item uses a 7-point rating scale plus a “not applicable” option, and all questionnaires are completed by infant caregivers.

#### Emotional Recognition

Pupillometry is evaluated using the Tobii X30-120 (Tobii AB), infant-compatible eye tracker, a high-precision, noninvasive, near-infrared device that emits low-intensity near-infrared light to form corneal reflection markers and captures real-time fluctuations in pupil diameter without disrupting spontaneous infant movement [32]. Stimuli are emotional facial photographs selected from the Edited Tromsø Infant Faces database, including happy, disgusted, and neutral facial expressions for emotional processing assessment [34]. All testing is standardized for 3-month-old full-term infants: each infant is held in a seated position in their primary caregiver’s arms at a fixed 60-cm viewing distance from the display screen. A pretest animated clip is presented at the center of the screen to draw the infant’s attention, followed by 5-point calibration to ensure accurate pupil signal recording. Each facial stimulus is displayed for 5 seconds, with an attention-grabbing central animation inserted between trials to sustain infant engagement [33]. If infants exhibit excessive crying or restlessness during calibration or stimulus presentation, data collection will be paused immediately, and caregivers will soothe the infant through rocking or gentle vocal comfort [35]. Testing can only resume when the infant calms down and voluntarily gazes at the central animation; persistent inconsolable distress will lead to session termination and rescheduling of a new test appointment to avoid invalid pupil data. Postrecording analysis extracts the mean difference in pupil dilation during viewing of emotional vs neutral faces as the primary marker of infant emotional recognition. Pupillometry that relies on near-infrared imaging is a well-validated, objective measure widely used to assess emotional processing in preverbal infants under 4 months of age.

#### Emotional Arousal

Galvanic skin response (GSR) [36] was captured by the Shimmer3 (Realtime Technologies Ltd [Shimmer Research]) portable physiological device. Medical-grade electrodes were attached to the infant’s plantar surface to record skin conductance signals at a sampling frequency of 50 Hz. Baseline conductance data were collected under quiet conditions, followed by presentation of standardized emotional facial stimuli selected from the Edited Tromsø Infant Faces database to evoke infant emotional responses. After signal acquisition, fixed amplitude thresholds were applied to eliminate motion artifacts during offline processing. Extracted parameters included skin conductance amplitude, response latency, and recovery time, which were used to reflect infants’ sympathetic nervous system activity and levels of emotional arousal. GSR measurement is a noninvasive physiological detection method commonly adopted for preverbal infants younger than 4 months.

### Secondary Outcomes

#### Mother-Infant Attachment Quality

This was measured using the Chinese version of the Maternal Postnatal Attachment Scale, validated by Huang et al [37]. This 30-item scale includes 3 dimensions: quality of attachment, absence of hostility, and pleasure in interaction. Higher scores indicate better attachment quality. The scale has good construct validity and internal consistency (Cronbach α=0.73, content validity index=0.93).

#### Salivary Oxytocin Levels

Saliva samples are collected from both mothers and infants at the 3-month visit, following a standardized protocol: collection between 8 to 10 AM, at least 1 hour after the last feeding, 30 minutes after a mouth rinse, and after 20 minutes of quiet mother-infant interaction [38,39]. Samples are centrifuged at 4000 r/min for 15 minutes at 4 °C, and oxytocin concentrations are measured via enzyme-linked immunosorbent assay (ELISA) [39]. Salivary oxytocin measured by ELISA has been validated as a reliable noninvasive biomarker in perinatal research.

### Variables and Measures

#### Demographic and Confounding Variables

Factors related to mothers include their age, where they live, level of education, financial situation, height and weight before and during pregnancy, number of previous births, method of delivery, postpartum sleep quality, symptoms of depression and anxiety, breastfeeding duration, and family support. For fathers, relevant aspects include age, job, educational background, participation in caring for the baby, tobacco and alcohol use, and paternal mental health status. For infants, relevant variables include gender, feeding pattern, primary caregiver, exposure to tactile stimulation, swimming lessons, passive exercise routines, daily outdoor activity duration, sleep patterns, temperament, and family rearing environment [45-47].

#### Measurement Tools for Confounding Covariates

General demographic information was collected using a structured questionnaire.

#### Maternal Confounding Factors

##### Depression and Anxiety Symptoms

The Chinese version of the Edinburgh Postnatal Depression Scale [48] is reliable and valid, with a sensitivity of 0.82 and a specificity of 0.86. The scale consists of 10 items, each scored from 0 to 3, yielding a total score ranging from 0 to 30. Score interpretation is defined as follows: 0 to 8 indicates no depression, 9 to 10 indicates mild depression, 11 to 12 indicates moderate depression, and 13 or higher indicates severe depression.

The Generalized Anxiety Disorder-7 scale [49] was used to assess participants’ anxiety status. This 7-item scale evaluates the severity and functional impairment of 7 anxiety-related symptoms over the preceding 2-week period. The scale has good reliability and validity, with a Cronbach α coefficient of 0.90. Each item is scored on a 4-point Likert scale ranging from 0 to 3, yielding a total score ranging from 0 to 21. Score stratification is defined as follows: 0 to 4 points for no anxiety, 5 to 9 points for mild anxiety, 10 to 14 points for moderate anxiety, and ≥15 points for severe anxiety, with higher total scores indicating greater severity of anxiety symptoms.

##### Postpartum Sleep Quality

The Pittsburgh Sleep Quality Index [50] was used to evaluate participants’ sleep quality over the preceding 1-month period. It consists of 19 self-rated items and 5 observer-rated items. The total Pittsburgh Sleep Quality Index score is computed by summing the component scores, with a score range of 0 to 21; higher scores indicate poorer sleep quality. The Cronbach α coefficients range from 0.83 to 0.84, and the split-half reliability coefficient is 0.824.

##### Family Support

Family support was evaluated using the Chinese version of the Perceived Social Support Scale [51]. The family support subscale (4 items) of the Perceived Social Support Scale was adopted to assess perceived family rearing-related support. Each item is scored on a 7-point Likert scale ranging from 1 (strongly disagree) to 7 (strongly agree); higher subscale scores indicate higher levels of perceived family support.

##### Parenting Stress

The Chinese version of the Parenting Stress Index–Short Form [52] was used to assess parenting-related stress among mothers. Higher total scores indicate greater perceived parenting pressure.

### Paternal Confounding Factors

Paternal mental health status was assessed using the Chinese version of the Generalized Anxiety Disorder-7 [53] to evaluate paternal anxiety symptoms, following the same scoring criteria described above.

### Infant Confounding Factors

#### Sleep Quality

The Chinese-language version of the Brief Infant Sleep Questionnaire [54], originally developed by Sadeh A in 2004 for infants aged 0 to 2 years, was used to assess infant sleep patterns. It includes items covering bedtime, sleep-onset latency, night awakenings, and daytime and nighttime sleep duration. The Brief Infant Sleep Questionnaire exhibits favorable reliability and validity (r>0.82) and is a widely-used practical tool for infant sleep assessment worldwide.

#### Infant Temperament

The Carey Temperament Scales, including the Early Infancy Temperament Questionnaire [55] and the Revised Infant Temperament Questionnaire [56], were adopted to evaluate infant attentional regulatory behaviors using 2 dimensions: persistence and distractibility. Persistence refers to the duration an infant sustains engagement in an activity and the impact of distraction on ongoing tasks. Distractibility describes the tendency of an infant’s attention to shift away from ongoing activities. The Early Infancy Temperament Questionnaire is suitable for infants aged 1 to 4  months, with internal consistency coefficients ranging from 0.43 to 0.76 across its dimensions.

#### Family Rearing Environment

The Family Rearing Environment Scale [57] for children aged 0 to 1 years was developed by He S et al in 2008 [58] to assess the family rearing environment of infants aged 0 to 1 years. This scale comprises 32 items and encompasses 4 factors: perception and cognition (13 items), emotional warmth (6 items), neglect and restriction (7 items), and human and computer interaction and feeding (6 items). It demonstrates favorable reliability and validity, with an overall Cronbach α coefficient of 0.918. Higher total scores indicate a more favorable family rearing environment for children aged 0 to 1 years.

### Quality Control

Training imparted to the researchers. The researchers are trained in the objectives of the research. They are also trained in the selection criteria for participants and guidelines for completing the scales. Further, they are trained in the procedures for measuring physiological indicators and handling unexpected events (eg, an infant crying). After passing the evaluation, they can take part in the study.

To ensure the quality of data collection, the researchers strictly adhere to the established inclusion and exclusion criteria for participant selection; liaise with the head of the research organization to solicit support and collaboration; conduct data collection in a quiet and pleasant environment to reduce the likelihood of strong emotional reactions in infants due to noise or an unfamiliar environment; and provide personalized assistance through a designated individual when mothers complete the questionnaire to address respondents’ queries promptly and ensure a consistent understanding of the questions.

For data entry, numbers are assigned to the collected questionnaires promptly, and a database is created. A 2-entry approach is used for data entry, followed by logical and consistency checks. The original data are checked for issues, and invalid questionnaires are rejected promptly to ensure data quality.

The saliva samples are labeled and stored in accordance with the relevant rules and kit instructions for oxytocin measurement, with appropriate quality control. It prevents sample mix-ups and degradation.

### Missing Data Handling

If less than 5% of the data for a variable are missing, complete-case analysis is used. If 5% to 20% of the data are missing, the missing values are imputed using appropriate imputation methods. However, variables with more than 20% missing data are excluded from the main analysis.

### Statistical Analysis Methods

All analyses are prespecified and directly aligned with the study hypotheses and research questions. Statistical analyses will be performed using SPSS (version 27.0; IBM Corp) and AMOS software (IBM Corp), with a 2-sided *P*<.05 considered statistically significant.

#### Descriptive Statistics

Continuous variables were presented as mean (SD) for normally distributed data, or median (IQR) for nonnormally distributed data. Categorical variables were presented as frequencies (percentages). This analysis provides a baseline characterization of the cohort and supports the evaluation of generalizability.

#### Univariate Screening

Independent-samples 2-tailed *t* test, 1-way ANOVA, Mann-Whitney *U* test, and chi-square test will be used to compare infant emotional development outcomes across categories of maternal PPF and each covariate. Variables with *P*<.10 in univariate analyses will be considered for adjustment in multivariable models [59]. This step identifies potential confounders for the main effect analysis.

#### Linear Association Analysis (Testing the Primary Hypothesis)

Spearman rank correlation will be used for preliminary bivariate exploration of the association between PPF scores and infant emotional development. Multiple linear regression models will then be constructed, with the infant emotional development composite score as the dependent variable and PPF severity as the independent variable, adjusted for key covariates. This analysis directly tests the primary hypothesis that higher PPF severity is independently associated with poorer infant emotional development.

#### Stratified Analysis by Parity

Stratified multiple linear regression analyses will be conducted to examine whether the association between maternal PPF and infant emotional development varies between primiparous (first-time mothers) and multiparous mothers. Separate covariate-adjusted regression models will be built within the 2 parity subgroups to generate corresponding effect size estimates.

#### Nonlinear Dose-Response Analysis (Extending the Primary Hypothesis)

A restricted cubic spline model will be fitted to examine the potential nonlinear relationship between continuous PPF score and infant emotional development, adjusted for covariates. A likelihood ratio test will be used to compare the nonlinear model with the linear model, and the fitted curve will be used to identify potential thresholds or inflection points. This analysis explores whether the association follows a dose-response pattern.

#### Chain Mediation Analysis (Tests the Secondary Hypothesis)

SEM with bias-corrected bootstrap (5000 resamples) will be applied to test the secondary hypothesis that salivary oxytocin levels and mother-infant attachment quality sequentially mediate the association between maternal PPF and infant emotional development. The mediation effect is considered statistically significant if the 95% CI does not include 0. This analysis directly evaluates the proposed mechanistic pathway.

### Ethical Considerations

This study was approved by the Ethics Committee of Nantong University (approval number: Tongda Lunshen [2025] number 70; date of approval: March 3, 2025). All procedures were designed and performed in strict accordance with the principles of the Declaration of Helsinki and the National Ethical Review Measures for Biomedical Research Involving Human Subjects of China.

#### Informed Consent

All eligible participants receive both verbal and written explanations of the study’s purpose, procedures, potential risks, and participants’ rights before enrollment. Paper-based written informed consent forms are signed by all participating mothers on behalf of both themselves and their infants prior to any study-related data or biological samples being collected. Participants are explicitly informed that they may withdraw from the study at any time without justification and that withdrawal will not affect their routine clinical care or follow-up services. Should participants withdraw from the study, all previously collected biological specimens will be destroyed, and their existing research data will be permanently excluded from the final analysis upon the participant’s request.

#### Infant Safety During Physiological Testing

During infant physiological assessment, including eye tracking and GSR measurement, researchers will comfort infants promptly when signs of distress appear. If an infant exhibits persistent, inconsolable crying that cannot be relieved by routine soothing measures, the entire assessment will be terminated immediately to minimize infant discomfort.

#### Standardized Saliva Specimen Processing

Upon completion of all scheduled study-related assessments, residual saliva biological specimens will be stored for a maximum of 2 years. After this retention period, all residual samples will be permanently destroyed in strict accordance with institutional ethical review board requirements. All disposable sampling supplies used for specimen collection will be categorized as biomedical waste and disposed of in accordance with local hospital biosafety and waste management regulations. No residual biological materials will be retained for additional research purposes without further ethical approval and renewed participant informed consent [23].

#### Privacy and Confidentiality

Each participant is assigned a unique, anonymous study ID upon enrollment. All personal identifiable information is stored separately from research data in a password-protected electronic system, with access restricted exclusively to authorized study personnel. All biological samples are labeled with study IDs only, with no direct link to personal information. Study results will be published only in aggregated form, with no individual participant identifiable.

#### Participant Compensation

No direct financial payment is provided for study participation. As a token of appreciation, participants receive age-appropriate infant educational toys and parenting guide brochures upon completion of each follow-up assessment. Participants with abnormal screening results (eg, high risk of postpartum depression or suspected developmental concerns) receive free referral to specialized clinical services.

## Results

Participant recruitment officially commenced in September 2025 and remains ongoing. By June 2026, a total of 180 mother-infant dyads had undergone eligibility screening. Of these, 146 dyads completed study enrollment, yielding a participation rate of 81.1%.

Among the 146 enrolled dyads, 12 withdrew before their infants reached the 3-month corrected-age follow-up window. Five dyads withdrew because the mothers declined to provide saliva samples; 4 dyads dropped out because the infants exhibited persistent crying and could not complete eye-tracking and GSR assessments; and 3 dyads withdrew owing to family schedule conflicts and voluntary withdrawal of informed consent.

After excluding these 12 dyads with early withdrawal, 134 dyads remained active in the cohort and awaited their scheduled assessment at 3 months corrected age. Follow-up assessments are carried out sequentially as enrolled infants reach this assessment time point.

By June 2026, 78 of these 134 active dyads had completed the full assessment battery at the 3-month follow-up. Assessment included behavioral questionnaires, physiological measurements via eye tracking and GSR, and saliva specimen collection for oxytocin testing.

A total of 4 dyads experienced participant attrition at the 3-month corrected-age assessment time point. Reasons included loss of telephone contact (n=2) and relocation without accessible contact information (n=2). The remaining 52 dyads are still under active observation because their infants have not yet reached the age required for follow-up.

Recruitment and follow-up activities will continue until the prespecified target sample size is achieved.

## Discussion

### Principal Findings

Infants’ development is influenced to some degree by adequate nutrition and physical safety [60]. When infants have a stable and safe environment, their emotional development is also affected [61]. Maternal fatigue after giving birth, which is quite common in the perinatal period, can interfere with an infant’s emotional development in several ways [62]. Nonetheless, current research in this field is somewhat scattered, and the mechanisms underlying this impact remain unclear [63]. This study aims to explore whether maternal PPF relates to the emotional development and whether oxytocin and maternal behaviors play a mediating role. The study has theoretical and practical significance [64,65].

This study develops a mediation model that connects PPF, oxytocin/maternal behaviors, and infant emotional development, thereby furthering the theoretical understanding of how maternal factors during the perinatal period affect infant emotional development [65,66]. By incorporating both physiological (oxytocin) and behavioral (maternal behaviors) measures into the study design, this study examines the complex and multifaceted effects of PPF on infant emotional development, thereby addressing a limitation of previous studies that focused on only 1 pathway [67].

The results of this study can provide a basic reference for maternal and child health services from a clinical perspective. By clarifying how PPF affects infant emotional development, the study may inform the implementation of targeted interventions in the near future [68]. Potential measures include improving the assessment of maternal fatigue postdelivery; offering psychosocial support to mothers experiencing fatigue, and helping mothers interact more effectively with their infants to minimize negative effects of PPF on infant emotional development [69]. This information can also be used to train maternal and child health care workers to recognize issues related to PPF and infant emotional development.

Salivary oxytocin serves as a peripheral biomarker that can be measured noninvasively and is suitable for paired sampling from mothers and infants. It avoids the discomfort associated with venous blood collection and allows repeated dynamic measurement [70]. However, this biomarker has inherent methodological constraints [25]. Salivary oxytocin concentrations can be affected by feeding time, oral residues, and oral cleaning procedures, even under standardized sampling protocols. In addition, peripheral salivary oxytocin concentrations cannot fully reflect central oxytocin activity because of the blood-brain barrier, and ELISA kits may introduce minor batch-to-batch differences in measurements [71]. In this study, we standardized sampling times, oral cleaning procedures, and laboratory centrifugation parameters to reduce interference. All analytical conclusions focus on relative differences between groups rather than absolute oxytocin values.

A 2-phase evaluation approach is proposed to examine the effects of PPF on infant emotional development. The first phase involves a preliminary assessment of maternal fatigue during the first week after delivery using the PFS to determine the severity and types of fatigue. The second phase involves assessing infant emotional development, maternal oxytocin levels, and maternal behaviors at 3 months postpartum using a mediational model. This approach is relatively simple to implement and may therefore be suitable for use in primary care and maternal and child services to improve early detection and intervention.

### Limitations

This study was conducted at a single site, with participants recruited from Nantong Maternity and Child Health Hospital and local community hospitals. The sample may be subject to selection bias, and the external validity of the findings should be confirmed through studies conducted at various centers.

The data on maternal fatigue after childbirth and lifestyle factors are self-reported and may be subject to recall bias. Future studies may employ objective measurements, such as activity and sleep monitors, to improve data collection.

The study followed participants for only 3 months after delivery; however, PPF may have cumulative effects on infant emotional development beyond this period. Future studies could include a longer follow-up period to examine the long-term effects of PPF on infant emotional development.

### Conclusions

The research examines the association between maternal PPF and infant emotional development using a prospective observational study. The mediation model will also examine the relationships among maternal and infant factors and family functioning. The findings are expected to deepen the understanding of how PPF affects infant emotional development. This study may provide a theoretical framework to guide interventions aimed at improving maternal and child health. The study also aims to create awareness of maternal fatigue after childbirth and improve maternal and child health care services to support infant emotional development.

## Supplementary material

10.2196/97348 Checklist 1 STROBE checklist.

## Abbreviations

ELISA: enzyme-linked immunosorbent assay
GSR: galvanic skin response
IBQ-R VSF: Infant Behavior Questionnaire-Revised Very Short Form
PFS: Postpartum Fatigue Scale
PPF: postpartum fatigue
SEM: structural equation modeling
STROBE: Strengthening the Reporting of Observational Studies in Epidemiology
